# How does team diversity relate to the willingness to collaborate with asylum seekers? It depends on the diversity dimensions investigated and boundary conditions

**DOI:** 10.1371/journal.pone.0266166

**Published:** 2022-03-28

**Authors:** Patrick F. Kotzur, Johannes Stricker, Ramona Fricke, Jonathan McPhetres, Bertolt Meyer

**Affiliations:** 1 Department of Psychology, Durham University, Durham, United Kingdom; 2 Department of Psychology, Heinrich Heine University Düsseldorf, Düsseldorf, Germany; 3 Institute for Economics, Private University of Witten-Herdecke, Witten, Germany; 4 Institute for Psychology, Technical University Chemnitz, Chemnitz, Germany; St John’s University, UNITED KINGDOM

## Abstract

The successful integration of asylum seekers into the labor market is among the most pressing issues of refugee-receiving countries. We construe co-workers’ willingness to collaborate with asylum seekers as a crucial factor for integration and investigate its antecedents. Linking Allport’s contact theory with team diversity theories, we propose that a work team’s diversity affects team members’ willingness to collaborate with asylum seekers. We thus investigated the effects of different facets of objective (national, migration background, age, and gender) and perceived diversity in work teams on team members’ willingness to collaborate with asylum seekers. In doing so, we also tested whether asylum seekers’ status in the team hierarchy (superior vs. colleague), task interdependence, and pro-diversity team norms moderate these effects. Multi-level regression analyses based on 470 participants nested in 106 teams showed that, overall, team diversity played a small role in explaining the willingness to collaborate with asylum seekers. Age diversity was negatively associated with the willingness to collaborate with asylum seekers, especially when asylum seekers were considered to take a post as a superior rather than a colleague. In teams with high task interdependence, migration background diversity and willingness to collaborate with asylum seekers were positively associated. Pro-diversity norms did not moderate team diversity effects. Overall, our findings demonstrate that team diversity can have beneficial, harmful, and no substantial consequences for the willingness to work with asylum seekers, depending on the considered type of diversity and boundary conditions.

## Introduction

Around the globe, an increase of forced migration since 2015 has raised the urgent question of how migration-receiving countries respond best to the associated challenges. The successful integration of the newcomers into the receiving countries’ labor market is among the most pressing related issues [1] as many refugees struggle with finding regular employment [2]. Besides other factors, such as the host countries’ labor market policies or asylum seekers’ professional qualifications, the integration of asylum seekers into the labor market also crucially depends on the willingness of potential coworkers to collaborate with asylum seekers at the workplace (see also [3]). However, as one German survey indicates, there are widespread concerns regarding the potential consequences related to the immigration of asylum seekers, such as increased competition in the job market [4]. These findings add fuel to the debate that increasing diversity may deteriorate intergroup relations and social cohesion more generally [5–7], and illustrate the need to investigate predictors of employees’ willingness to collaborate with asylum seekers at work.

In this study, we propose that employees’ team properties–specifically, the team’s diversity—affects team members’ willingness to collaborate with asylum seekers. This is because the team constitutes employees’ immediate social environment at the work place with profound effects on team members’ perceptions and motivations [8]. By investigating the effects of work team diversity on employees’ willingness to collaborate with asylum seekers, we attempt to contribute to several streams of literature. Firstly, we connect research on work team diversity, which has primarily investigated the diversity-performance link (see [7], for a review), with research on the societal consequences of diversity such as the perception of social groups [6, 9–11].

Secondly, we contribute to research on intergroup contact (e.g., [12, 13]) by proposing that experiences in diverse work teams generalize to being more open towards other diversity types as well.

Thirdly, most studies conducted in organisations have focused on the effects of objective diversity (e.g., the divergence on demographic characteristics such as age, ethnicity, or gender, cf. [14–17]). Studies that have been conducted in other contexts shows, however, that the *perception* of diversity may also play a prominent predictor of intergroup attitudes, and thus intentions towards groups [9–11]. Consequently, we contribute by exploring both the effects of facets of *objective* and *perceived* team diversity on the willingness to collaborate with asylum seekers.

Fourthly, researchers have suggested boundary conditions governing the effects of team diversity (see, e.g., [18]). Adding to this research, we also propose and test potential moderators of the influence of diversity on the willingness to cooperate with asylum seekers. The proposed moderators are based on Allport’s [19] original formulation of the intergroup contact hypothesis and were adapted to the work context: cooperation within the contact situation (task interdependence of team members), support by local norms (pro-diversity team norms), and status (anticipated position of the asylum seeker within the work team).

### Intergroup contact effects on attitudinal outcomes

The intergroup contact hypothesis proposes that positive interactions between members of different social groups ameliorate intergroup relations [19]. Allport [19] formulated four conditions under which intergroup contact unfolds its beneficial effects on attitudes: equal status, common goals, intergroup cooperation, and support of authorities, norms, and customs. A large body of empirical evidence supports these postulations [20, 21], while the conditions for positive effects of intergroup contact are rather conducive than indispensable [21].

Many studies have subsequently applied the intergroup contact hypothesis to the contextual level (e.g., [22, 23]). According to these authors, individuals within contextual units with larger shares of outgroup members are provided with more opportunities for intergroup contact experiences (e.g., [24]). Consequently, these individuals should have more favorable attitudes towards the respective outgroup than individuals in units with lower shares of outgroup members [12, 23]. Indeed, contextual outgroup exposure has been linked to opportunities for intergroup mixing, which can lead to positive intergroup encounters and more favorable attitudes [6, 25]. For instance, the ethnic composition in social units has been linked to higher levels of acceptance of immigrants or ethnic minorities ([6, 12, 22, 23, 25–29] but see, e.g., [24, 30]).

Thus far, research in this domain has been rarely linked to contact in professional settings (for an exception, see [6]). However, researchers have argued that smaller social units, such as one’s immediate work context, lend more meaningfulness to individuals than larger, commonly-studied units, such as one’s neighborhood or county [13]. This is because most people negotiate their everyday relationships in small-scaled contexts [13]. Therefore, we aim to add to this literature by examining diversity effects in the work context. In doing so, we focus on work teams, i.e., groups of people perceiving themselves as such due to their work on a shared goal [14–16], since teamwork is the most common form of organizational collaboration [31].

Another branch of intergroup contact research suggests that the beneficial effects of intergroup contact are not limited to the groups involved in the encounter [32–35]. That is, the effect of intergroup contact on attitudes is not restricted to the respective outgroup present in the contact situation, but also affects attitude levels towards groups not involved; a process that has been coined the secondary transfer effect [33]. Empirical studies support this postulation [36–39]. Work in this realm suggests a generalization gradient, in a way that the transfer effect is stronger for outgroups that are similar or overlapping to the ones involved in the intergroup encounter [33, 40]. To our knowledge, none of the existent studies has tested secondary transfer effects on attitudes towards potential colleagues in work settings.

Intergroup contact requires some degree of diversity among those involved. Diversity can be defined as differences between individuals on any attribute that may lead to the perception that another person is different from oneself, such as nationality, migration background, age, or gender [cf. 14–16]. Following the intergroup contact hypothesis and Blau’s [41] proposition that diversity serves as a facilitator of integrative group processes, we argue that diversity in teams is equivalent to regular collaborative intergroup contact in the work setting. Consequently, higher levels of diversity in work teams should be linked to more intergroup contact, unlike in other settings, where cross-group contact can also be avoided (e.g., [42]). Hence, higher levels of diversity should lead to more positive attitudes towards the involved groups. Through the secondary transfer effects, this process should expand to a reduction in prejudice towards asylum seekers, expressed in an increased willingness to collaborate with them in one’s work team.

Although objective diversity is a necessary precondition for *intergroup* contact to occur, some researcher argue that the *perception* of diversity may be a more important determinant [9–11]. We contribute by exploring both the effects of commonly-studied facets of *objective* diversity–nationality, migration background, age, and gender–alongside general *perceived* team diversity on the willingness to collaborate with asylum seekers:

*H1a*: *Objective team diversity in nationality*, *migration background*, *age*, *and gender is positively associated with the willingness to collaborate with asylum seekers in teams*.
*H1b*: *Perceived team diversity is positively associated with the willingness to collaborate with asylum seekers in teams*.

### Moderators of the relationship between team diversity and willingness to collaborate with asylum seekers

The direct and indirect effects of intergroup contact are highly sensitive to contextual factors (for a review, see, e.g., [33]). Hence, this study further investigated the conditions that may influence the effects of work team diversity on collaboration intentions towards asylum seekers via secondary transfer effects of varying types of diversity. Such conducive conditions have already been introduced by Allport [19], as specified above. To our knowledge, these conditions have not been tested with regard to work team diversity. We are also unaware of empirical studies investigating Allport’s [19] optimal conditions as moderators of secondary transfer effects. Therefore, we followed recent calls for empirical tests of these optimal conditions [43]: equal status (operationalized as status of the asylum seeker in the team hierarchy), cooperation (operationalized as task interdependence), and support by authorities, laws, and custom (operationalized as pro-diversity team norms) by considering them as moderators of the relationship between team diversity and willingness to collaborate with asylum seekers. In doing so, we acknowledge that common goals are already an integral part of the definition of teams (e.g., [14–16], and thus refrained from putting this condition to test.

#### Equal status–status of the asylum seeker in the team hierarchy

Allport [19] considered status differences as an important factor inhibiting the emergence of positive intergroup relations. Status can be defined as “any characteristic, such as gender or skin color, on which people are hierarchically ordered as higher or lower” ([44], p. 59). As such, status serves as a fundamental organizer of social perception in contemporary societies [45]. Social status is associated with competence perceptions related to respect and the outgroups’ perceived capability and agency [45]. Task-related qualities, such as competence, are typically required and desired in the work context [46].

In organizational contexts, perceived status and competence of social groups meet hierarchical structures within teams, which are themselves associated with perceptions of status and competence (e.g., team leader/supervisor roles vs. assistant roles). Role congruity theories [47, 48] suggest that perceived incongruity between the social status of a social group and of a position can result in a perceived inadequacy for members of that social group to take on such positions. Whereas leadership positions are associated with high status, esteem, competence, and power, majority members typically attribute low social status and low competence, and high need for assistance to asylum seekers [49, 50]. These perceptions are likely to affect performance expectations [51] and thus (un)suitability perceptions for high competence positions. Additionally, majority members oppose status challenges from low-status groups [44, 52]. Accepting asylum seekers in a higher status position than one’s own can be seen as such a challenge. Thus, asylum seekers’ anticipated position in the team hierarchy (same status position vs. superior status position) should have a moderating impact on the relationship between team diversity and one’s willingness to work with asylum seekers.

*H2a*: *The positive association between objective diversity in nationality*, *migration background*, *age and gender and willingness to collaborate with asylum seekers will be stronger for same-status positions (colleague) compared to superior status positions (supervisor)*.
*H2b*: *The positive association between perceived team diversity and willingness to collaborate with asylum seekers will be stronger for same-status positions (colleague) compared to superior status positions (supervisor)*.

#### Cooperation–task interdependence

Group processes have a significant impact on team-related outcomes [53]. One determining factor of intra-team cooperation is task interdependence, i.e., the extent to which team members have to rely on each other to accomplish their work [54].

Within the intergroup contact literature, it has been suggested that intergroup cooperation in interdependent tasks is beneficial for positive intergroup attitudes to emerge [19]. This contention has received widespread empirical support across a diverse array of team contexts and compositions (e.g., [55–57]). Although a minimal level of cooperation among team members, who–by definition–share common goals, can be seen as given in teams, levels vary as a function of task properties. Task interdependence is causally related to cooperation [58] and is contingent for positive diversity effects to emerge [59, 60]. Tasks with higher task interdependence require more frequent communication and sharing of knowledge or resources to achieve group outcomes [60]. Consequently, higher task interdependence enhances contact and cooperation levels between team members, which directly translates into higher levels of intergroup contact in diverse teams compared to homogenously composed teams. Thus, we argue that task interdependence in diverse teams, compared to homogenous teams, is related to higher levels of intergroup contact, which in turn should lead to stronger contact effects.

*H3a*: *Higher task interdependence increases the positive association between objective team diversity in nationality*, *migration background*, *age*, *and gender and willingness to collaborate with asylum seekers*.
*H3b*: *Higher task interdependence increases the positive association between perceived team diversity and willingness to collaborate with asylum seekers*.

#### Pro-diversity team norms (support by authorities, laws, and customs)

Allport [19] suggested that contexts welcoming cross-group interaction are beneficial for positive intergroup attitudes to emerge. Recent studies suggest that social norms in a given context welcoming or rejecting intergroup interactions strongly influence prejudice levels (e.g., [22, 61, 62]. Whereas descriptive norms refer to “the perception of what most people do” ([63], p. 202), injunctive norms refer to “norms that characterize the perception of what most people approve or disapprove” ([63], p. 202). Both norms play a vital role in coloring intergroup relations. For instance, an upward shift of positive attitudes towards refugees and other migrant groups in Canada just after President Trudeaux (an arguably more liberal and immigration-welcoming authority) took office can be explained by a shift of descriptive and injunctive norms [64]. In line with these observations, individuals without personal intergroup encounters express more positive intergroup attitudes in contexts where intergroup contact is the norm [61]. Typically, highly prejudiced individuals show a greater reduction of prejudice in contexts where intergroup encounters are the norm [22]. Moreover, research has shown a positive trend between the effect sizes of intergroup contact on intergroup attitudes and the emergence of norms valuing equality between groups in the US [62]. Consequently, we expect that pro-diversity work team norms will moderate the relationship between diversity and willingness to collaborate with asylum seekers.

*H4a*: *Higher pro-diversity team norms increase the positive association between objective team diversity in nationality*, *migration background*, *age*, *and gender and willingness to collaborate with asylum seekers*.
*H4b*: *Higher pro-diversity norms in teams increase the positive association between perceived team diversity and willingness to collaborate with asylum seekers*.

## Method

### Sample and Procedure

Low risk studies, like ours, do not require a formal clearance from an internal review board in Germany, where this study has been conducted. All procedures were performed in accordance with the ethical guidelines of the Deutsche Gesellschaft für Psychologie (German Society for Psychology). Data was collected online, and consent given in written form (per check box).

We recruited 470 participants (*M*_*age*_ = 34.36, *SD*_*age*_ = 11.92; 55.74% female, 41.06% male, 3.2% missing; 69.79% with professional qualification, 28.94% without professional qualification, 1.27% missing; 93.83% German nationality; 4.04% non-German nationality, 2.13% missing; 16.80% with migration background, 79.36% without migration background, 3.8% missing) nested in 106 project and work teams (*M*_*Team size*_ = 10.57, *SD*_*Team size*_ = 8.49) in Germany in spring 2016. Thus, whereas the gender ratio in our sample was balanced and there was substantial variability in participants’ age, our data comprised participants of mostly German nationality, and participants without migration background. The participating teams were tasked with a wide range of different assignments, and stemmed from diverse sectors (19.4% public sector; 27.7% voluntary work; 0.03% police; 33.8% private sector; 10.6% other; 5.5% missing) and organizations (ranging from large retailers to small project teams). As an incentive, teams were provided with a summary of study results upon request.

This data was collected in a larger collaborative data collection effort. Two further empirical articles were based on the collected data [65, 66]. These articles addressed entirely different research questions (focusing on the relation of team composition and team member’s mental health). Hence, both the research question and constructs used are unique to this article and do not constitute dual publication. We have uploaded the data set that contains all variables relevant to this project as well as a data transparency table on an open science framework page, https://osf.io/8v6tm/.

### Measures

#### Objective team diversity

Objective diversity in teams is often quantified using the standard deviation of continuous attributes (e.g., age [67]) and the Blau Index for categorical variables (e.g., gender). The Blau Index quantifies the probability that two randomly selected team members would have different attributes [41]. Following the literature on objective diversity, we calculated the level of dispersion in the teams using the standard deviation for age, and Blau Index for gender, migration background, and nationality.

#### Perceived team diversity

To measure perceived diversity, we adapted a scale from Meyer, Shelma, and Schermuly [68]. On a scale from 1 = *completely disagree*, to 5 = *completely agree*, α = .81, participants indicated their agreement to four items (e.g., “Regarding its composition, my team is diverse”).

#### Willingness to collaborate with asylum seekers

We measured the individuals’ willingness to cooperate with asylum seekers on a self-developed scale ranging from 1 = *completely disagree*, to 5 = *completely agree*, with three items for each of the two status dimensions (colleague vs. superior). The same-status subscale included the following items: “I can hardly imagine an asylum seeker as a colleague in my team” (reverse coded), “I approve of an asylum seeker becoming part of my team”, “I can imagine well working together with an asylum seeker in my team”, α = .87. The higher status subscale included the following items: “I can hardly imagine an asylum seeker as a leader of our team”, “I approve of an asylum seeker as a leader of our team”, “I can imagine well working together with an asylum seeker as the leader of our team”, α = .90.

#### Task interdependence

To measure task interdependence, we adapted a scale from Van der Vegt and Janssen [69]. On a scale from 1 = *completely disagree*, to 7 = *completely agree*), participants indicated their agreement to four items, such as: “I have a one-person job; it is not necessary for me to coordinate or cooperate with others” (reverse coded), α = .75.

#### Pro-diversity team norms

We used a four-item scale by Meyer & Schermuly [70] to measure individual pro-diversity beliefs. The scale ranged from 1 = *does not apply at all*, to 5 = *applies fully*. Reliability analyses revealed inadequate internal scale consistency, α = .29. In a subsequent principal component analysis, two items (“Solving complex problems requires teams with different backgrounds and experiences”and “I prefer to work with people I consider similar to myself” (reverse coded)) loaded on a common factor, which we used in subsequent analyses. Since Cronbach’s Alpha is dependent on scale length, internal consistency of the 2-item version of the scale was acceptable, α = .51 [71]. Next, we group-mean centered individuals’ responses to aggregate them the team-level, representing pro-diversity norms within the team context.

#### Control variables

Besides the demographic variables age, gender, and the type of organization (1 = public sector; 2 = voluntary work; 3 = police; 4 = private sector; 5 = other), we also controlled for personal intergroup contact experiences with asylum seekers and individual-level pro-diversity beliefs. For the former, we used an adapted version of the one-item measure by Barlow et al. [72]. On a scale from 1 = *never*, to 5 = *often*, we asked: “How often do you generally have contact with asylum seekers?”. For the latter, we used the participants’ deviation from the team’s group mean-centered pro-diversity norm score.

## Results

### Preliminary analyses

All analyses were performed using the R version 1.2.1 statistical environment [73] using the lme4 package [74]. The syntax for our main analyses can be found on this project’s open science framework page, accessible under https://osf.io/8v6tm/. Means, standard deviations, and bivariate correlations between measures on the individual level are summarized in Table 1. ICCs were calculated for the unconditional model including only the two random effects. The cross-level interaction accounted for about 40% of variance in willingness to work with asylum seekers (ICC = .399). Team ID accounted for about 29% of variance in willingness to work with asylum seekers (ICC = .285) suggesting that there is ample variance at both levels. Following recommendations, we started with the maximal random effects structure for each model given our sample size and reduced it from that point as necessary [75]. As random-intercept random-slope models did not converge, all reported models are random-intercept models.

**Table 1:** Means, standard deviations, and correlations between variables on the individual level.

		M	SD	1	2	3	4	5	6	7	8	9
1	Intention to collaborate with asylum seekers as colleagues	4.13	0.92									
2	Intention to collaborate with asylum seekers as superiors	3.68	1.14	.77***								
3	Diversity beliefs	4.17	0.69	.37***	.30***							
4	Task interdependence	4.48	1.20	.11*	.11*	.24***						
5	Perceived diversity	4.91	1.17	-.10*	-.12*	.10*	.05					
6	Migration background (1 = yes, 2 = no)	1.83	0.38	.00	-.04	.05	-.06	.04				
7	Gender (1 = female, 2 = male)	1.58	0.49	.14**	.05	.08	.04	.05	.05			
8	Age	34.36	11.92	-.18***	-.24***	.06	-.04	.15**	.15**	-.02		
9	Team size	10.57	8.49	.15**	.19***	.14**	.12*	-.04	-.05	-.01	-.23***	
10	Intergroup contact	2.35	1.03	.14**	.09	.17***	.01	.03	.04	.03	.01	.01

*Note*. * *p* < .05,

** *p* < .01,

*** *p* < .001.

Nationality is not displayed in this table because it is a multi-categorical variable, meaning that means, standard deviations, and correlations of this measure are not meaningfully interpretable on the individual level.

### Main analyses

The results of all main analyses are summarized in Table 2. According to the first set of hypotheses, objective team diversity in nationality, migration background, age, gender (H1a), and perceived diversity (H1b) are positively associated with the willingness to collaborate with asylum seekers. To test these hypotheses, we computed linear mixed-effects models with random intercepts for subject and team. Data were nested in two levels (individuals within teams) which included the control variables team size, team type (dummy coded, with public sector as reference category), and pro-diversity norms on the team level, as well as age, gender, intergroup contact, mean-centered task interdependence, and mean-centered diversity beliefs on the individual level (Model 1).

**Table 2:** Parameters in the estimated models.

	Model 1		Model 2		Model 3		Model 4		Model 5	
	*b*	95% CI	*b*	95% CI	*b*	95% CI	*b*	95% CI	*b*	95% CI
Intercept	1.96	[0.69, 3.23]	2.21	[0.78, 3.65]	2.14	[0.71, 3.58]	2.52	[0.79, 4.25]	1.50	[-2.21, 3.61]
**Team Level**										
Team size	-0.00	[-0.01, 0.01]	0.00	[-0.01, 0.02]	0.00	[-0.01, 0.02]	0.00	[-0.01, 0.02]	-0.00	[-0.02, 0.01]
Team type: voluntary work	0.29	[-0.06, 0.63]	0.00	[-0.38, 0.39]	0.00	[-0.38, 0.39]	-0.04	[-0.44, 0.36]	-0.01	[-0.41, 0.38]
Team type: police	-2.15	[-2.80, -1.50]	-2.24	[-2.89, -1.59]	-2.23	[-2.88, -1.59]	-2.26	[-2.92, -1.61]	-2.31	[-2.97, -1.65]
Team type: private sector	0.04	-0.26, 0.34]	-0.00	[-0.30, 0.30]	-0.00	[-0.30, 0.30]	-0.02	[-0.33, 0.29]	-0.02	[-0.33, 0.29]
Team type: other	0.36	[-0.04, 0.76]	0.37	[-0.02, 0.77]	0.37	[-0.03, 0.77]	0.40	[-0.01, 0.80]	0.40	[-0.01, 0.80]
Pro-diversity norms	0.55	[0.24, 0.86]	0.51	[0.18, 0.83]	0.51	[0.18, 0.83]	0.50	[0.16, 0.83]	0.70	[-0.28, 1.67]
National diversity (Blau index)			-0.16	[-1.19, 0.87]	-0.10	[-1.18, 0.98]	3.00	[-0.22, 6.21]	7.49	[-9.21, 24.19]
Migration background diversity (Blau index)			0.44	[-0.16, 1.04]	0.41	[-0.22, 1.03]	-1.57	[-3.40, 0.26]	-3.31	[-9.16, 2.45]
Age diversity (standard deviation)			-0.04	-0.07, -0.01]	-0.03	[-0.06, 0.00]	0.00	[-0.07, 0.08]	0.04	[-0.26, 0.34]
Gender diversity (Blau index)			0.20	[-0.34, 0.74]	0.13	[-0.44, 0.70]	-0.09	[-1.95, 1.78]	3.55	[-3.09, 10.20]
**Individual Level**										
Age	-0.01	[-0.02, -0.01]	-0.01	[-0.02, -0.00]	-0.01	[-0.02, -0.00]	-0.01	[-0.02, -0.00]	-0.01	[-0.02, -0.00]
Gender	-0.04	[-0.21, 0.14]	-0.00	[-0.18, 0.17]	-0.00	[-0.18, 0.18]	-0.05	[-0.22, 0.13]	-0.02	[-0.20, 0.16]
Intergroup contact with asylum seekers	0.10	[0.02, 0.19]	0.11	[0.03, 0.20]	0.11	[0.03, 0.20]	0.11	[0.02, 0.19]	0.11	[0.02, 0.20]
Task interdependence	0.02	[-0.05, 0.09]	0.01	[-0.06, 0.08]	0.01	[-0.06, 0.08]	-0.03	[-0.25, 0.20]	0.01	[-0.06, 0.08]
Diversity beliefs	0.34	[0.21, 0.47]	0.35	[0.22, 0.48]	0.35	[0.22, 0.48]	0.35	[0.22, 0.48]	0.35	[0.22, 0.48]
Perceived diversity			-0.04	[-0.11, 0.03]	-0.04	[-0.12, 0.04]	-0.13	[-0.41, 0.16]	-0.00	[-0.82, 0.82]
**Intra-Individual Level**										
Status of asylum seeker within team	-0.44	[-0.51, -0.37]	-0.44	[-0.51, -0.37]	-0.31	[-0.53, -0.09]	-0.44	[-0.52, -0.37]	-0.44	[-0.51, -0.37]
**Cross-Level Interactions**										
Status of asylum seeker within team x nationality diversity					-0.13	[-0.81, 0.55]				
Status of asylum seeker within team x migration background diversity					0.08	[-0.32, 0.48]				
Status of asylum seeker within team x age diversity					-0.03	[-0.04, -0.01]				
Status of asylum seeker within team x gender diversity					0.13	[-0.24, 0.51]				
Status of asylum seeker within team x perceived diversity					-0.01	[-0.07, 0.06]				
Task interdependence x nationality diversity							-0.69	[-1.34, -0.03]		
Task interdependence x migration background diversity							0.44	[0.06, 0.83]		
Task interdependence x age diversity							-0.01	[-0.03, 0.00]		
Task interdependence x gender diversity							0.08	[-0.32, 0.48]		
Task interdependence x perceived diversity							0.02	[-0.04, 0.08]		
Pro-diversity norms x nationality diversity									-1.86	[-5.94, 2.22]
Pro-diversity norms x migration background diversity									0.91	-0.49, 2.32]
Pro-diversity norms x age diversity									-0.02	[-0.09, 0.05]
Pro-diversity norms x gender diversity									-0.80	[-2.39, 0.79]
Pro-diversity norms x perceived diversity									-0.01	[-0.20, 0.19]

Public service was reference category for team type; female was reference category for gender; status as a colleague was reference category for asylum seekers’ status.

Next, we ran a model in which we additionally included all measures of team diversity (nationality diversity, migration background diversity, age diversity, gender diversity on the team level, and mean-centered perceived diversity on the individual level) simultaneously. Only the 95% confidence intervals of age diversity excluded zero, indicating a significant effect. Contrary to our expectations, higher age diversity was associated with *less* willingness to collaborate with asylum seekers, *b* = -0.04, 95% CI [-0.07, -0.01]. Therefore, our results did not support Hypotheses 1a and 1b.

**Fig 1:**
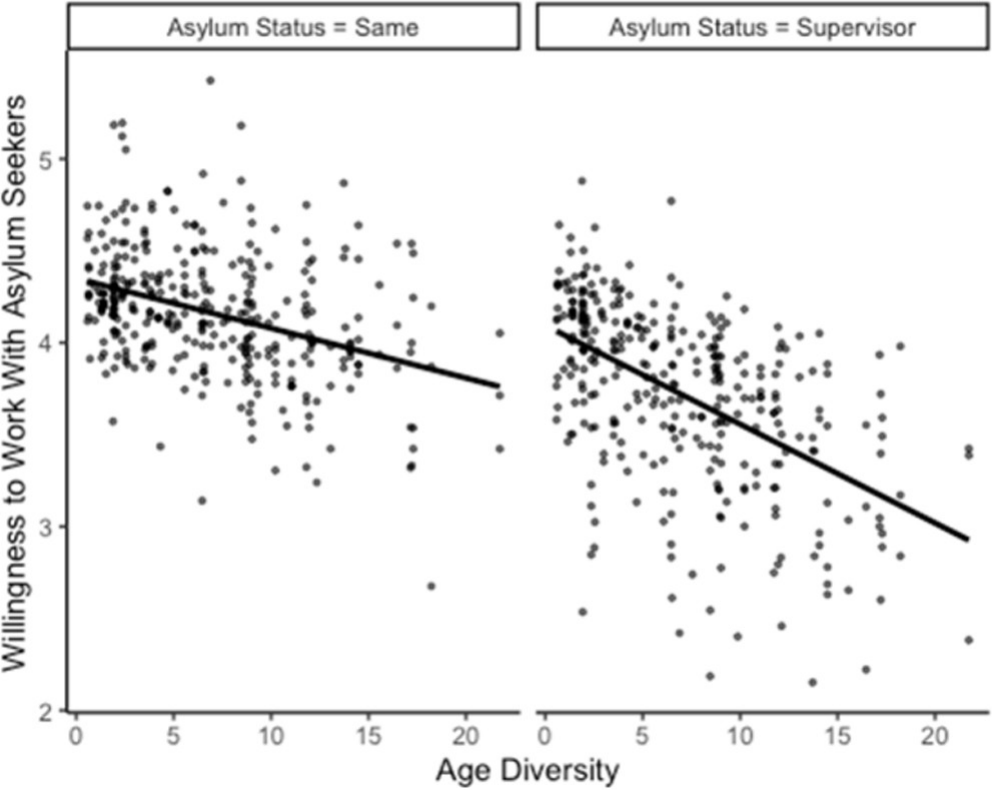
Significant interaction effect of status of the asylum seeker in one’s work team (same status vs. supervisor role) and age diversity on willingness to collaborate with asylum seekers, controlled for control variables.

According to the second set of hypotheses, the positive association between objective diversity in nationality, migration background, age, and gender (H2a) and willingness to collaborate with asylum seekers, as well as perceived diversity and willingness to collaborate with asylum seekers (H2b), should be stronger for same-status positions (colleague) compared to superior status positions (supervisor). Hence, we included interaction effects between all diversity measures and the status of asylum seekers in Model 3. Only the 95% confidence interval of the interaction effect between age diversity and asylum seekers’ status on the willingness to collaborate with asylum seekers excluded zero, *b* = -0.03, 95% CI [-0.04, -0.01]. To facilitate the interpretation of the significant interaction, we plotted willingness to collaborate with an asylum seeker as a function of age diversity and status position following the guidelines provided by Preacher, Curran, and Bauer ([76], see Fig 1). Contrary to our hypothesis, age diversity was *more negatively* related to the willingness to collaborate with asylum seekers as a superior, *b* = -0.05, *p* < .001, compared to as a colleague, *b* = -0.03, *p* = .068. Overall, the results neither supported H2a, nor H2b.

**Fig 2:**
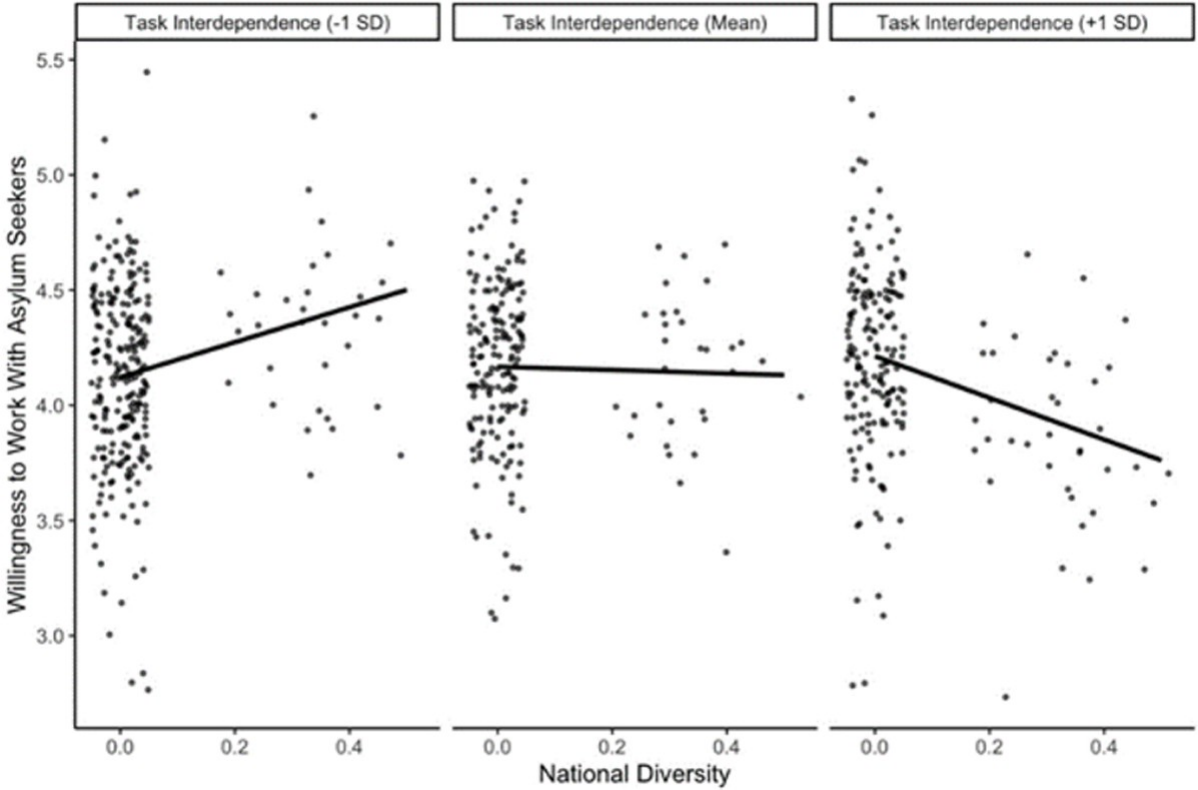
Significant interaction effect of task interdependence and nationality diversity on willingness to collaborate with asylum seekers controlled for control variables.

**Fig 3:**
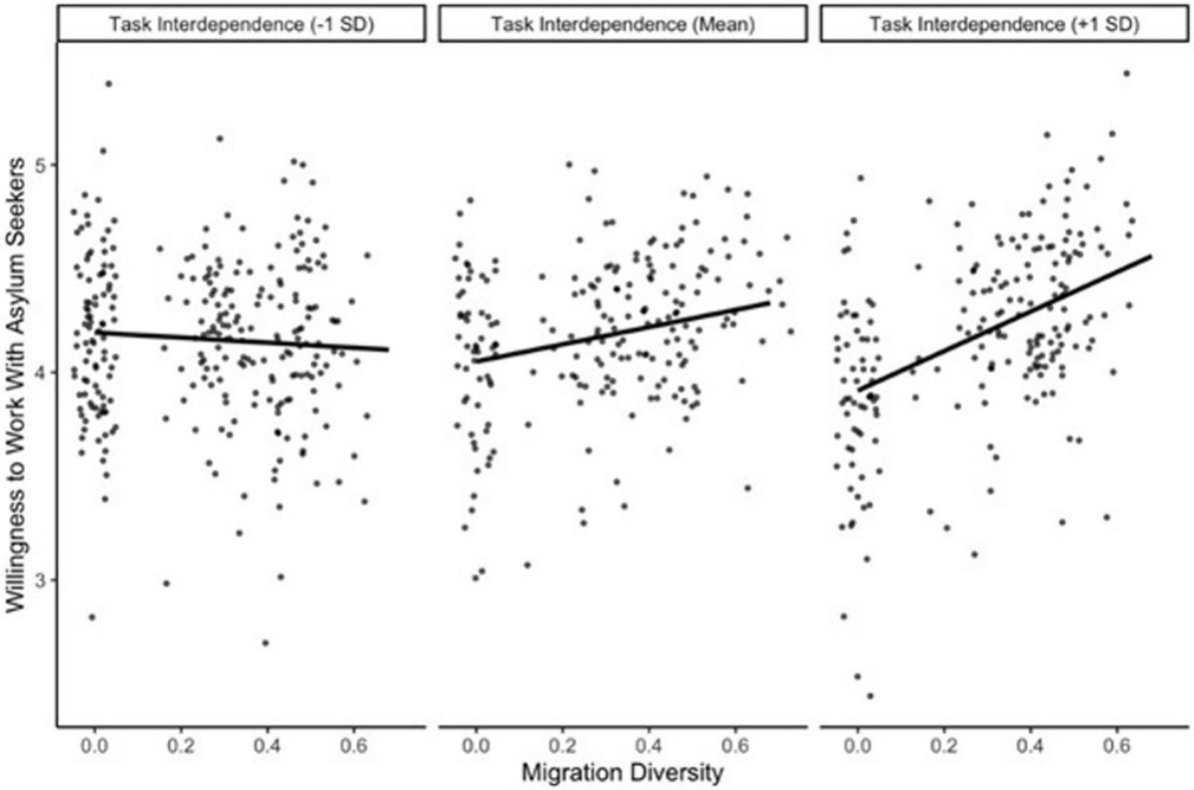
Significant interaction effect of task interdependence and migration background diversity on willingness to collaborate with asylum seekers, controlled for control variables.

According to the third set of hypotheses, higher task interdependence increases the positive association between objective team diversity in nationality, migration background, age, and gender (H3a) and willingness to collaborate with asylum seekers, as well as perceived diversity (H3b). Thus, in Model 4, we dropped the interaction effects of Model 3, and included interaction effects between all diversity measures and task-interdependence on the willingness to work with asylum seekers. Only the 95% confidence intervals of the interaction effects of nationality diversity and task interdependence, *b* = -0.69, 95% CI [-1.34, -0.03], as well as migration background diversity and task interdependence, *b* = 0.44, 95% CI [0.06, 0.83], excluded zero. Again, significant interactions are illustrated in Figs 2 and 3 following Preacher et al. [76].

Unexpectedly, none of the simple slopes involving nationality diversity were significant. In teams with low task interdependence, migration background diversity and willingness to work with asylum seekers were not significantly related, *b* = -0.12, *p* = .758. In teams with high task interdependence, migration background diversity and willingness to collaborate with asylum seekers were positively associated, *b* = 0.95, *p* = .014. Thus, the pattern of results showed partial support for H3a when considering migration background diversity, whereas H3b was not supported.

According to the fourth set of hypotheses, higher pro-diversity norms in teams increase the positive association between objective team diversity in nationality, migration background, age, and gender (H4a) and willingness to collaborate with asylum seekers, as well as perceived diversity and willingness to collaborate with asylum seekers (H4b).

Thus, in Model 5, we dropped the interaction effects of Model 4 and included interaction effects between all diversity measures and team-level pro-diversity norms on the willingness to work with asylum seekers instead. None of the 95% confidence intervals of the interaction effects excluded zero, indicating that both H4a and H4b were not supported by our data.

As the relationship between objective and perceived diversity and willingness to work with asylum seekers was not moderated by team-level pro-diversity norms, we conducted further exploratory analyses with individual-level (group-mean centered) diversity beliefs. Results of these analyses are reported in the online supplementary materials.

## Discussion

The successful integration of newcomers in the labor market of receiving countries is among the most pressing issues associated with increased forced migration worldwide [1]. Acknowledging that this integration can only be successful if employees are willing to collaborate with asylum seekers at work, we investigated the effects of objective and perceived diversity in work teams on employees’ collaboration intentions with asylum seekers. We tested our predictions in Germany, a country that was a major refugee-receiving country within Europe since 2015 [77].

In line with the intergroup contact hypothesis, we expected higher levels of team diversity to lead to more positive attitudes towards the respective outgroup, which in turn should make members of diverse teams more open to other newcomers as well (H1a, H1b). However, contrary to our expectations, team composition was not significantly associated with willingness to collaborate with asylum seekers overall. Only age diversity was significantly related to the willingness to collaborate, yet, contrary to our expectations, *negatively* so. As such, our results are at odds with research suggesting that diverse contexts are on average more welcoming of newcomers (e.g., [6, 12, 22, 23, 25–29]). Although the body of literature that finds positive diversity effects is large, prior work has found that diversity may also have negative effects on the openness to newcomers (e.g., [5]) and suggested boundary conditions under which diversity may unfold more likely conducive or detrimental effects (see, e.g., [18]). Thus, one explanation might be that the moderators might have been aligned in such a way in our sample that the net effect of diversity was neutral or, in the case of age diversity, negative, which might have not been the case in previous studies we used to deduce our first set of hypotheses from.

We propose an additional post-hoc explanation for the negative effect of age diversity. Highly diverse teams typically consist of members that are both young and old. Age is generally associated with more conservative views and prejudice towards outgroups [78–80]. Consequently, older team members may, on average, express stronger reservations to collaborate with asylum seekers. This is supported by the negative correlation between age and willingness to work with asylum seekers (see Table 1). Younger team member may be aware of the likelihood that their older team members might be less accepting of asylum seekers than others. As a consequence, those younger participants, who may otherwise be more accepting of asylum seekers in their teams, might have reservations to welcome asylum seekers into their age diverse team [81]. Irrespective of this speculation, our finding indicates that age diversity as a facet of team diversity can have harmful consequences for the willingness to work with asylum seekers.

Adding to the debate of moderators, we also proposed and tested moderator candidates of the influence of team diversity on the willingness to collaborate with asylum seekers. Based on Allport’s [19] original formulation of the intergroup contact hypothesis, we tested to what extent the status of an asylum seeker in the team hierarchy (equal status vs. higher status), task interdependence (cooperation), and pro-diversity team norms (support by authorities, laws, and customs) qualified the relationship between team diversity and willingness to collaborate with asylum seekers.

As for task interdependence (Allport’s cooperation), we had expected that higher task interdependence increases the positive association between team diversity and willingness to collaborate with asylum seekers (H3a, H3b). As expected, in teams that reported higher task interdependence, migration background diversity and willingness to collaborate were more closely and positively associated than in teams that reported lower task interdependence. This finding supports the idea that positive interdependent cooperation in diverse settings fosters more welcoming attitudes towards newcomers [19, 55–57, 59, 60]. Although a significant interaction effect with nationality diversity did emerge, none of the simple slopes reached statistical significance. Additionally, a closer inspection of this diversity variable revealed that our sample was highly skewed towards national homogeneity, reducing the trustworthiness of these results. Future research with a more nationally diverse sample may produce more robust findings.

We could not replicate the moderating effect of task interdepence on the association between migration background diversity and willingness to work with asylum seekers across other diversity dimensions. One explanation might be that–in line with work on the secondary transfer generalization gradient [33, 40]–the other diversity categories could be too dissimilar from those of asylum seekers. Consequently, daily interactions in work settings with people with migration background might make people especially more welcoming towards outgroups that have a migration history when team member need to rely on each other to get their work done. Overall, this findings demonstrates that the migration background diversity facet of team diversity can have beneficial effects on the willingness to work with asylum seekers, yet only in teams with high task interdependence.

As for the status of the position of the asylum seeker in the team hierarchy (Allport’s equal status), we had expected accepting asylum seekers in a higher status and power position than one’s own can be challenging. Thus, we expected that the *positive* association between diversity and willingness to collaborate with asylum seekers should be stronger for same-status positions (colleague) compared to superior status positions (supervisor; H2a, H2b). Contrary to our expectations, the status of the position of the asylum seeker did not play an important role in qualifying the effects of diversity on willingness to work of asylum seekers most of the time. When it did, results were not in line with our expectations either. This is, because the *negative* association between age diversity and willingness to collaborate with asylum seekers was stronger for an asylum seeker as a superior compared to an asylum seeker as a colleague. Our speculations about the particularities of age diversity elaborated on above might help explain these, on the first glance surprising, findings: Whereas older team members of highly age diverse teams may express their reservations to collaborate with asylum seekers, younger team member that may have had more positive views towards asylum seekers may have adjusted their answers to account for this circumstance, rejecting asylum seekers in a superior position more strongly than in a same-status position.

As for the moderator pro-diversity norms (Allport’s support by authorities, laws, and customs), we expected that higher pro-diversity norms in teams increase the positive association between team diversity and willingness to collaborate with asylum seekers (H4a, H4b). Among other things, these postulations were based on the idea that welcoming norms are one of the important ingredients for positive intergroup interactions in diverse teams to emerge [22, 61, 62], which in turn should make them more welcoming contexts for newcomers. This, however, was not the case.

Overall, our study provided unique insights into the relationship between various facets of team-level diversity and individual-level willingness to work with asylum seekers in teams in a number of ways. As such, we added to the literature that proposes and debates factors that determine the successful integration of asylum seekers at the workplace [3]. Our findings demonstrate that, most of the time, team diversity plays no central role in shaping the willingness to work with asylum seekers. Also, we did not find evidence for the idea that the perception of diversity might systematically play a more prominent role in shaping intergroup attitudes than objective diversity, as prior literature suggested [9–11]. Nonetheless, our results indicate that team diversity can have both harmful and beneficial consequences for the willingness to work with asylum seekers, depending on the kind of diversity considered, and its boundary conditions.

We recruited a large number of teams across different industries to address research questions of high academic, social, and political relevance during a time in which the need for successful integration of asylum seekers in the labor market was particularly high (i.e., in 2016, during the heydays of the so-called European “refugee crisis”). Given this circumstance, we advise future research to replicate our study to test the robustness of our findings in different time and country settings. Moreover, we used a cross-sectional design, which limits our ability to draw causal conclusions. Although alternative causal relationships are implausible for most of the variables we included in our analyses (e.g., it is rather unlikely that willingness to work with asylum seekers predicts diversity in teams), we encourage future research to account for this limitation. Lastly, our research revealed a number of unexpected relationships between the constructs we investigated that invited us to speculate about them. We want to stress the need for future research to follow up on these findings to advance our understanding of what kind of team diversity under which conditions has positive, negative, or no substantial effects on willingness to work with asylum seekers.
